# Zinc Oxide Nanoparticles from Waste Zn-C Battery via Thermal Route: Characterization and Properties

**DOI:** 10.3390/nano8090717

**Published:** 2018-09-12

**Authors:** Rifat Farzana, Ravindra Rajarao, Pravas Ranjan Behera, Kamrul Hassan, Veena Sahajwalla

**Affiliations:** Centre for Sustainable Materials Research and Technology (SMaRT@UNSW), School of Materials Science and Engineering, UNSW Sydney, NSW 2052, Australia; r.rajarao@unsw.edu.au (R.R.); p.behera@student.unsw.edu.au (P.R.B.); kamrul.hassan@unsw.edu.au (K.H.); veena@unsw.edu.au (V.S.)

**Keywords:** waste Zn-C batteries, Zinc-oxide, nanoparticles, optical properties, sustainability

## Abstract

Disposable batteries are becoming the primary sources of powering day-to-day gadgets and consequently contributing to e-waste generation. The emerging e-waste worldwide is creating concern regarding environmental and health issues. Therefore, a sustainable recycling approach of spent batteries has become a critical focus. This study reports the detail characterization and properties of ZnO nanoparticles recovered from spent Zn-C batteries via a facile thermal synthesis route. ZnO nanoparticles are used in many applications including energy storage, gas sensors, optoelectronics, etc. due to the exceptional physical and optical properties. A thermal treatment at 900 °C under an inert atmosphere of argon was applied to synthesize ZnO nanoparticles from a spent Zn-C battery using a horizontal quartz tube furnace. X-ray diffraction (XRD), selected area electron diffraction (SAED) and X-ray photoelectron spectroscopy (XPS) results confirmed the formation of crystalline ZnO nanoparticles. Field emission scanning electron microscopy (FESEM) and transmission electron microscopy (TEM) analysis confirmed that the size of synthesised ZnO particles were less than 50 nm and mainly composed of sphere shaped nanoparticles. Synthesized ZnO exhibited BET surface area of 9.2629 m^2^/g and showed absorption of light in the UV region. Excitation of ZnO by UV light showed photoluminescence in the visible range. This study will create an opportunity for potential applications of ZnO nanoparticles from spent batteries and will benefit the environment by reducing the volume of e-waste in landfills.

## 1. Introduction

Zinc oxide (ZnO) is a material of special interest due to its wide band gap of ~3.3 eV, *n*-type semiconducting properties, unique optical behaviour and excellent chemical and thermal stability [1]. Due to its exceptional properties, ZnO is used in many applications such as electronics, optoelectronics, sensors, lasers, solar cells, photo catalysts, pharmaceutical, energy harvesting applications [2,3,4]. It is a promising material for many optoelectronic applications like ultraviolet lasers, light emitting diode, thin film transistors etc. The properties of ZnO nanomaterials can be improved by doping, changing their size, shape, chemical composition and surface area [5,6]. Several methods including chemical vapour deposition, arc discharge, sol-gel, hydrothermal, microbial route, oxidation process, etc. have been employed to synthesise ZnO nanostructures, using conventional materials like zinc acetates or nitrates precursor [7,8,9,10]. Most of these techniques require complicated facilities and expensive precursors or chemicals which ultimately hinder low-cost and large-scale fabrication of ZnO nanostructures [3,11]. Thus, synthesis of ZnO nanoparticles from waste sources could provide an attractive and sustainable solution for the future. The closure of many mines and deficit in raw material has pushed the price of zinc (Zn) by 60% in 2016 and expected to be $2900/ton in 2019 [12]. An alternative Zn/ZnO source is crucially needed to address price issue and ensuring availability of ZnO for various vital applications. Therefore, recovery of ZnO nanoparticles from spent Zn-C battery via a facile thermal route has the potential to provide economical and sustainable benefits.

E-waste is one of the fastest growing waste streams due to the decrease in the life span of electronic gadgets and change of product design/technology at regular intervals [13]. Batteries, as energy storage device, are widely used and have contributed significantly to the total e-waste. The demand for battery is forecasted to grow annually by 7.7% and reach US$ 120 billion in 2019 [14]. It is estimated that in Australia approximately 350 million batteries including stand alone or embedded in products are consumed every year. Handheld batteries comprise 98% (on count basis) of these consumed batteries including Zn-C battery which contribute 19%. As the Zn-C battery is a nonchargeable handheld battery type, a huge number of spent Zn-C battery are added to the e-waste stream and mostly landfilled [15]. Zn-C batteries are mainly used for alarm clocks, remote controls, radios etc. and contain large amounts of manganese (Mn), and Zn. Recycling of waste batteries is crucial in order to recover valuable metals and also to prevent threat to the ecology and human health. Recovering valuable metals from waste batteries can reduce the demand for virgin metals and in conserving the vast energy utilized in the process to extract metals from their respective ores. In recent years, several hydrometallurgical approaches using acidic or alkaline dissolution to recover mainly Mn and Zn are reported [16,17]. Few studies have reported recovery of ZnO via hydrothermal/chemical routes [18]. However, additional solvent extraction and electrochemical steps are required to recover value-added materials via hydrometallurgy. Pyrometallurgical routes require high energy consumption but less operative steps which facilitate simpler routes to recover value-added materials from waste. Industrial pyrometallurgical process Bartec uses very high temperature (1550 °C) to recover Zn, and Mn alloy [16]. Pyrometallurgical studies reported mainly to recover metals like Zn and Mn. Besides, this study details recovery of ZnO and its properties which implies the prospect of ZnO for potential applications [16,19].

In our group, a number of studies have been reported to recover value-added materials including nanomaterials from e-waste [20,21]. Recently, the thermal route ‘Thermal nanosizing to synthesise ZnO and MnO nanoparticles simultaneously from waste zinc-carbon (Zn-C) battery has been reported [22]. This study focuses on the characterization and properties of synthesised ZnO nanoparticles via thermal nanosizing. Characterization and analysis of properties of ZnO were investigated by XRD, XPS, Raman, Brunauer-Emmett-Teller (BET), FESEM, TEM, ultraviolet-visible spectroscopy (UV-Vis) and photoluminescence spectroscopy (PL) analysis. Synthesized ZnO nanoparticles were spherical in shape and within 50 nm size. As-synthesized ZnO nanoparticles showed absorption of UV light at wavelength of 388 nm and photoluminescent in visible range could be potentially useful for optical applications. This approach also can be an economical route to produce ZnO nanoparticles and effective in reducing waste battery in landfills.

## 2. Experimental

### 2.1. Materials and Methods

**Figure 1:**
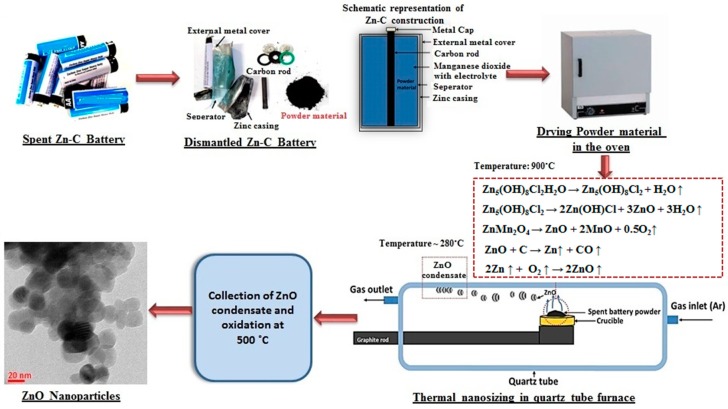
An overall flow diagram to synthesize ZnO from spent Zn-C battery which includes schematic representation of typical Zn-C battery construction, dismantled battery fraction, experimental set up and synthesized ZnO nanoparticles.

Waste Zn-C battery was manually dismantled to separate the external metal cover, zinc casing, separator, metals caps, carbon rod and powdered materials. Powdered materials were packed between the carbon rod (positioned centrally, acts as a conductor) and zinc casing (acts as a negative electrode). The powdered materials are the positive electrode and contains mainly manganese dioxide and zinc chloride as electrolyte. A schematic representation of Zn-C battery and dismantled fractions are shown in Figure 1. The powder materials (positive electrode), wetted with electrolyte was used as raw material to synthesize ZnO nanoparticles. The obtained powder materials were dried in the oven at 90 °C for 2 h to remove moisture. Horizontal Quartz tube (length: 1000 mm, diameter: 45 mm) furnace was used for this experiment which also includes a gas supply system and a graphite rod to hold the sample. The carrier gas argon was passed at a flow rate of 1 L/min from the gas inlet of the tube throughout the experiment. The dried powder was loaded on the ceramic crucible and placed on the graphite rod. The graphite rod was then pushed into the furnace where the temperature was 900 °C and kept there for 1 h. The greyish material which was agglomerated to the quartz tube surface at the low temperature zone (temperature 280 ± 20 °C) near the gas outlet was separated and collected. The obtained powder material was then kept at 500 °C in an air atmosphere to remove hydroxide impurities followed by subsequent analysis. An overall flow diagram to synthesise ZnO nanoparticle from waste Zn-C battery is shown in Figure 1.

### 2.2. Characterization Methods

Phase analysis of the raw material and synthesised nanoparticles were analysed by X-ray diffraction, XRD (Philips, PANalytical X’Pert Pro multipurpose, Australia) using CuKα radiation of 45 kV and 40 mA as the radiation source. Samples were scanned in the 2θ range from 10° to 100° diffraction angles, under the step size of 0.026° with 1° slit and 10 mm mask. Phase identification was done by using Xpert High Score Plus software (version 4.7). X-Ray Photoelectron Spectroscopy, XPS (ESCALAB250i, Thermo Scientific, UK) was conducted with standard conditions, mono-chromated AlKα (energy 1486.68 eV), 150 W (12 mA and 13 kV). Spot size was 500 micrometers, 90° photoelectron take off angle and 100 eV, 20 eV for survey scan and region scans respectively. Field emission scanning electron microscope, FESEM (FEI Nova NanoSEM 450) and transmission electron microscope, TEM (JEOL-1400) along with selected area diffraction (SAD) techniques are applied to analyse morphology of the ZnO nanoparticles. ZnO particles were coated with Platinum (Pt) for FESEM to make the sample conductive. ZnO particles were suspended in ethanol, dispersed ultrasonically to separate individual particles, and two drops of the suspension deposited onto holey-carbon coated copper grids for TEM analysis. BET surface area was conducted by N_2_ physisorption on a Micromeritics Tristar II Plus absorption analyser from relative pressure (p/p_0_) 0 to 1. The samples (~0.5 g) were dried at 110 °C in the oven and then degassed for at least 3 h at 150 °C under vacuum prior to analysis. The absorption spectrum was obtained by a computer interfaced UV-Visible spectrometer (PerkinElmer Lambda 35). Synthesized ZnO was dispersed in ethanol and ultrasonicated and used for optical absorption spectra using 1 mm pathlength cuvette from 600 to 200 nm wavelength. Photoluminescence (PL) analysis was conducted using Renishaw inVia Raman spectrometer using near UV lasers of excitation wavelength 325 nm coupled with an optical microscope having 15× objective lens.

## 3. Results

**Figure 2:**
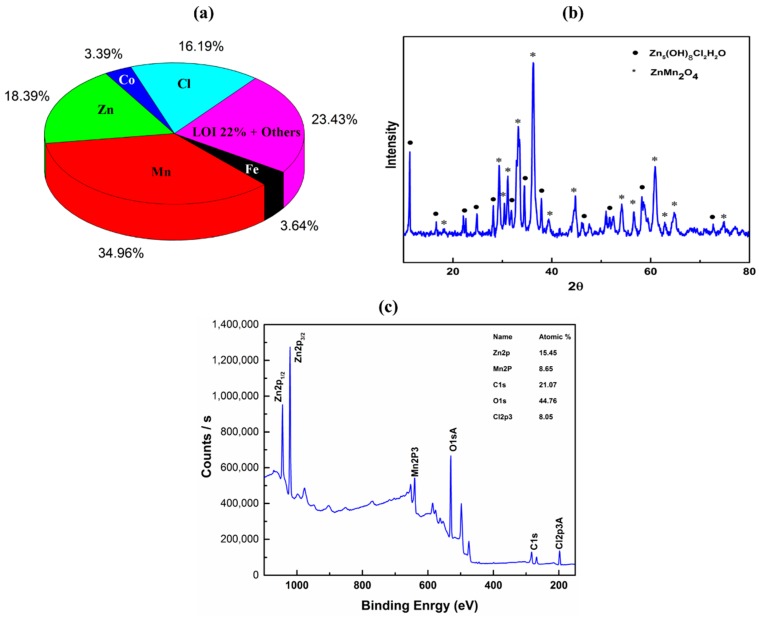
(**a**) Chemical composition (wt. %) in oxide form and (**b**) XRD analysis (**c**) XPS analysis of spent battery powder used to synthesize ZnO nanoparticles.

X-ray fluorescence spectroscopy, XRF of spent battery powder is shown in Figure 2a. Zn (18.39 wt. %), Mn (34.96 wt. %) in oxide form, Cl (16.19 wt. %) were the major elements in the analysis and other minor oxides include Fe, Co, Ca, Si, Cr, Ni, K etc. XRD analysis of the waste battery powder is shown in Figure 2b, confirm the presence of mainly ZnMn_2_O_4_ (hetaerolite) and Zn_5_(OH)_8_Cl_2_H_2_O (simonkolleite) phases. Formation of hetaerolite and simonkolleite is in agreement that the major elements in the battery are Zn, Mn, Cl, Oxygen in XRF analysis data and available literature of Zn-C battery. XPS analysis of spent battery powder showed Zn, Mn, Cl and O which is also in good agreement with materials analysis by XRF and XRD. Presence of C in XPS can be attributed to the presence of carbon in battery and/or carbon from carbon rod during battery dismantling.

**Figure 3:**
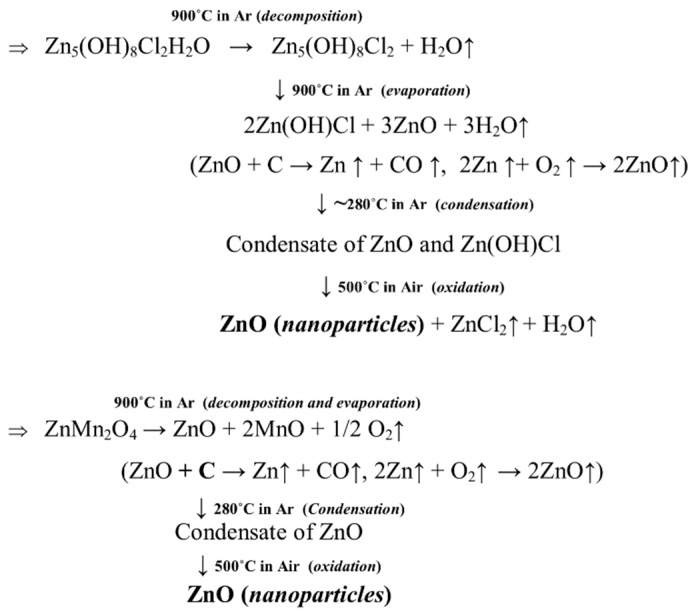
Formation mechanism of ZnO nanoparticles from waste Zn-C battery.

Simonkolleite starts to decompose upon heating at 900 °C under an inert atmosphere leaving a mole of water, and further prolonged heating decomposes it to ZnO and Zn(OH)Cl (zinc chloride hydroxide) [23]. ZnO reduced to Zn by carbothermal reduction (C present in battery mixture) and produced Zn vapor. Zn vapor again formed ZnO through in-situ oxidation [24]. In addition, at 900 °C ZnMn_2_O_4_ also decomposes to ZnO which also reduced to Zn vapor and oxidised into ZnO in the gas phase and finally condensed as ZnO nanoparticle [25,26]. ZnO formation may occur through decomposition of ZnO into Zn vapor and oxygen and recombination depending on different parameters [25,26]. Major elements such as Mn, along with other impurity elements (like Fe, Si) which were present in the battery powder, remain in the residue which was discussed in detail in a previous study [22]. Further oxidation of collected powder under air atmosphere at 500 °C removed hydroxide impurity leaving only ZnO as the residue. The thermal nanosizing mechanism [22] to synthesise ZnO nanoparticles from waste Zn-C battery is shown in Figure 3 and is also displayed in Figure 1.

**Figure 4:**
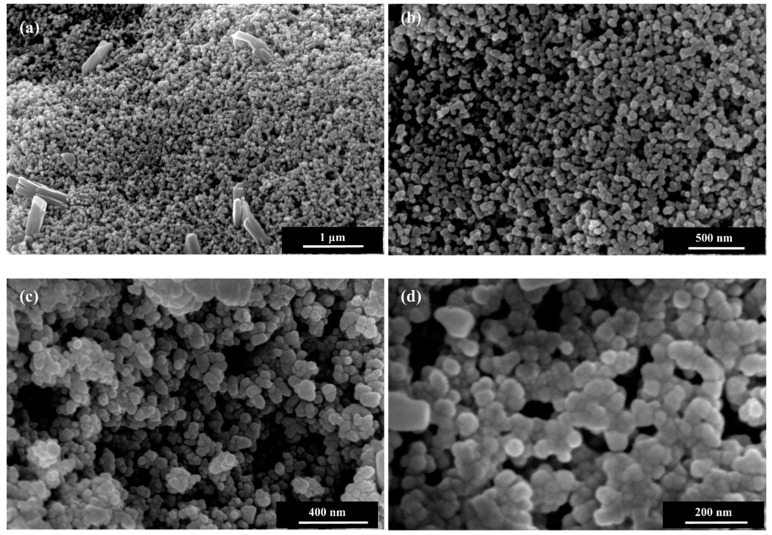
FESEM images of ZnO nanoparticles at different magnification (**a**) 65,000×; (**b**) 120,000×; (**c**) 200,000×; and (**d**) 350,000×.

**Figure 5:**
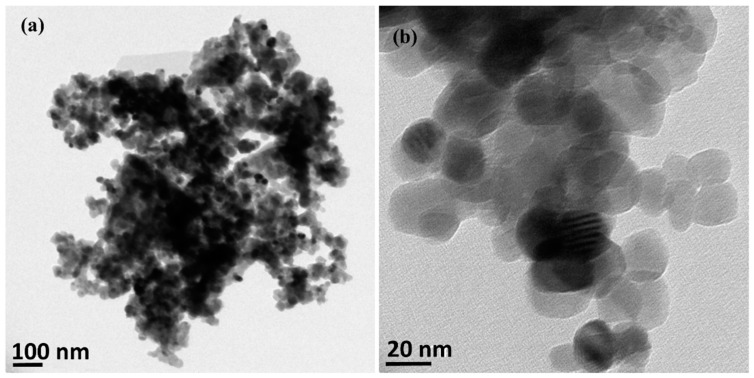
(**a**) Low and (**b**) High resolution TEM images showing the morphology of as-synthesized ZnO nanoparticles.

As-synthesised ZnO nanoparticles were analysed using FESEM, TEM, XRD and XPS analysis. The synthesised powder was white gray in colour, which was similar to the colour of ZnO obtained using conventional synthesis from zinc acetates or nitrates. Representative low and high magnification FESEM images (65,000× to 200,000×) of synthesised ZnO nanoparticles are shown in Figure 4. The microstructure observed at low and high magnification FESEM confirmed that the recovered ZnO particles are in the nano range and composed of sphere-shaped nanoparticles. The morphology of the nanoparticles was almost similar in shape and the size was within 50 nm. Particles were mainly homogenously distributed though aggregation of particles was observed in some areas of the SEM images. The TEM image in Figure 5 also confirmed the formation of nanoparticles in spherical shape and the represented size of the nanoparticles was in the range of 10–40 nm.

**Figure 6:**
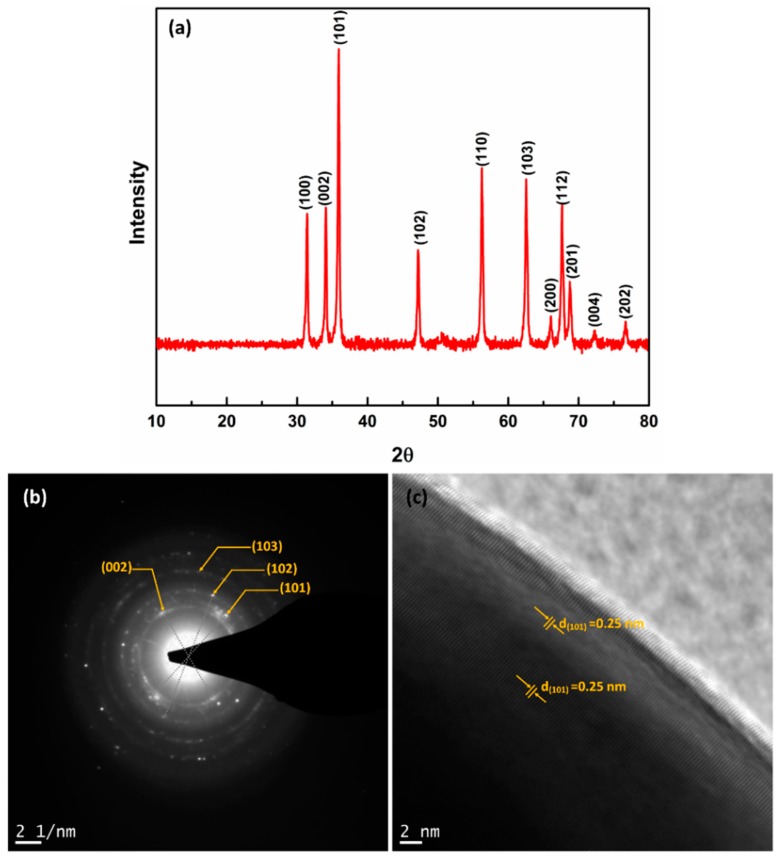
(**a**) XRD pattern (**b**) SAED pattern and (**c**) lattice fringes of as-synthesized ZnO nanoparticles.

XRD peaks of synthesised ZnO nanoparticles shown in Figure 6a, at 2θ ~ 31.67°, 34.31°, 36.14°, 47.40°, 56.52°, 62.73°, 66.28°, 67.91°, 69.03°, 72.48° and 76.96° were assigned to (100), (002), (101), (102), (110), (103), (200), (112), (201), (004) and (202) of ZnO nanoparticle (Reference Code: 03-065-3411). The peaks of as-synthesised ZnO particles indicated the nanocrystalline nature and matches the pure ZnO standard peaks [27]. XRD analysis confirmed the low/no impurity of the obtained ZnO as there were no other characteristic impurities peaks. The lattice parameters for hexagonal crystal structure (a = b= 0.328 nm and c = 0.522 nm) and d spacing values were calculated for major peaks using Bragg’s equation which matches with observed reference pattern are shown in Table 1. Particle sizes using different Miller indices were estimated by following the Debye-Scherrer formula (Equation (1)), where D is the crystallite size, k is shape factor (k = 0.9), β is the full width at half maxima (measured by Gaussian fit using Origin), λ is the wavelength of X-ray (Cukα) and θ is the diffraction angle. The average particle size ~27 nm is in agreement with the observed particle size from HRTEM image (Figure 5).

(1) D = kλ/βcosθ

The Bragg reflection of as-synthesized ZnO nanoparticles were also measured by SAED pattern (Figure 6b). Polycrystalline nature of ZnO nanoparticles was confirmed by the bright spots, making up rings coming from the Bragg reflection from each crystallite. The crystallite distances were well matched with (002), (101), (102) and (103) which are in agreement with XRD result. The presence of lattice fringes of ZnO nanoparticles in Figure 6c, represents the crystalline nature and distance between the fringes was 0.25 nm which corresponds to the dominant (101) plane and matched with the calculated d value from XRD.

XPS results are shown in Table 2. Atomic % of Zn2p3, 29.96% and O1s, 43.77% were the highest and represented the formation of ZnO. Low atomic % of impurities, such as Cl2p3, Si2p, Ca2p3A, K2p3 etc. were also observed which could attributed to impurity during ZnO collection and can be removed by dissolution by acid if high purity material is required. C1s at 284.8 eV was used as binding energy reference therefore could be attributed to adventitious hydrocarbon. The highest atomic % at binding energy peaks at 1021.87 eV corresponding to Zn2p3 confirmed the presence of ZnO. O1sA with atomic percentage 26.53% at 530.36 eV, could be assigned to oxidized metal ions specifically Zn-O present in the ZnO lattice. O1sB at 531.6 eV with 13.63 atomic % is attributed to loosely bound oxygen (O^2−^ ions) on the surface or oxygen deficient region within ZnO matrix [28]. O1sC with small atomic % of 3.61, at 532.7 eV should be assigned to OH species of absorbed H_2_O molecules onto the surface of the ZnO nanoparticles [29].

**Figure 7:**
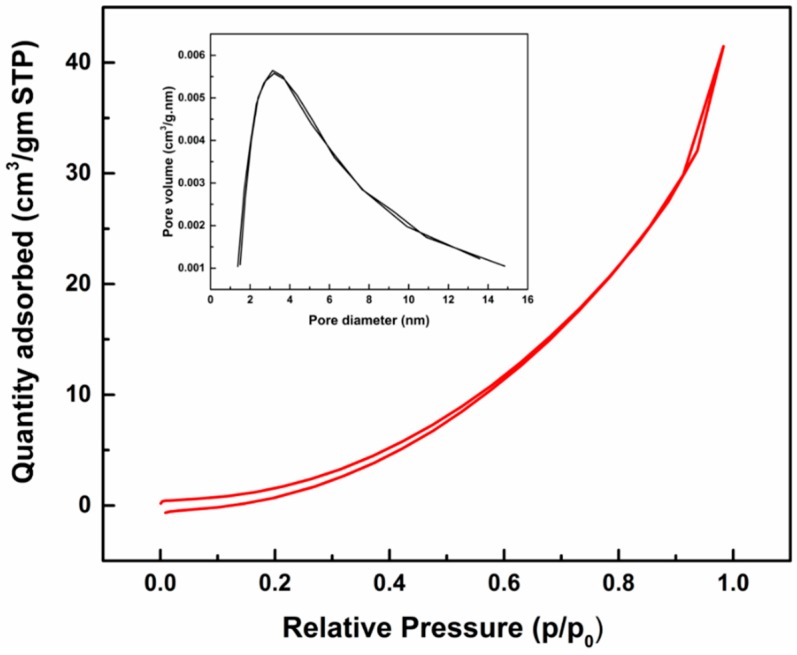
Nitrogen adsorption-desorption isotherms and Barret-Joyner-Halenda (BJH) pore size distribution (inset) of as-synthesised ZnO nanoparticles.

Properties of as-synthesized ZnO nanoparticles from spent Zn-C battery were observed by BET analysis, UV-Vis and Photoluminescence spectroscopy. Specific BET surface area is an important microstructural parameter of ZnO particles, which depends on the geometrical shape and porosity of the particles. BET surface area and porosity parameters are given in Table 3. Synthesized ZnO showed BET surface area of 9.2629 m^2^/g which is comparable with the literature [30,31]. The average BJH pore diameter was ~5 nm which demonstrates that the ZnO nanoparticles comprise of micro and mesopores as per IUPAC definition. A type III isotherm (Figure 7) was observed with no/minor hysteresis loop. The BJH pore size distribution of the ZnO nanoparticle (inset of Figure 7) shows that major pores were within 5 nm and larger pores also coexist [22].

**Figure 8:**
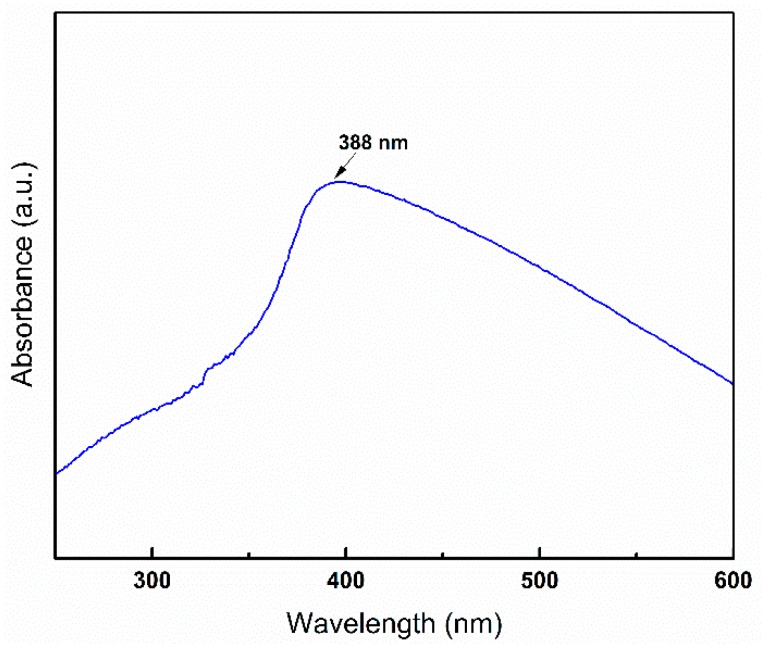
UV-Visible absorption spectrum of synthesised ZnO nanoparticles.

The room temperature UV-Vis optical absorption spectrum for the ZnO nanoparticles is shown in Figure 8. The excitonic absorption peak of the ZnO nanoparticles was observed at ultraviolet region ~388 nm (3.2 eV), which originates from band edge absorption of synthesized ZnO and is in agreement with literature data [32,33]. The absorbance value is dependent on the various factors such as size of particles, flaws or deformities in grain structure, and oxygen deficiency [34,35]. The lower band gap value of as-synthesized ZnO compared to bulk ZnO 3.3 eV (370 nm), could be attributed to the presence of oxygen vacancy defects. The particle size of the ZnO as a function of peak absorbance wavelength was measured by effective mass model by the following mathematical formula (Equation (2)) [36]. Here r is the particle radius and λ_p_ is the peak absorbance wavelength in nm. The particle size was around 12 nm which is in broad agreement with TEM particle size. The absorption of ZnO nanoparticles in the UV region demonstrates the potential applications where UV absorption is required.

(2) $$r\;=\;\frac{-0.3049\;+\;\sqrt{-26.23012\;+\;\frac{10240.72}{\lambda_{p}}}}{-6.3829\;+\;2483.2/\lambda_{p}}$$

**Figure 9:**
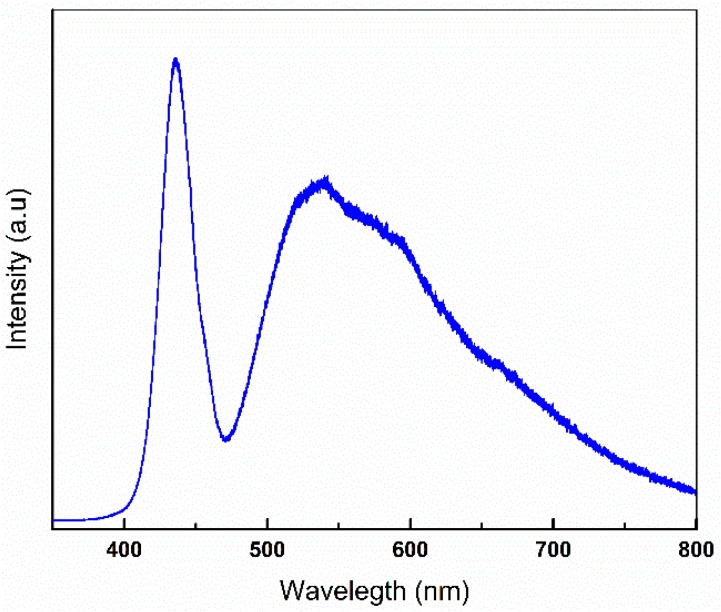
Photoluminescence spectra of synthesised ZnO nanoparticles.

The optical properties of synthesized ZnO was also studied by PL spectroscopy and the spectra is shown in Figure 9. The excitation energy 3.8 eV (325 nm) which is higher than the bulk ZnO (3.3 eV) and as-synthesized ZnO (3.2 eV) band gap energy was used so that an electron in the valence band could directly be excited to the conduction band and to the deep levels within the band gap was possible. Room temperature PL spectra of synthesized ZnO showed emission band of visible range at ~439 nm corresponding to blue emission and at ~538 nm corresponding to the green emission. These peaks are found in literature and could be associated with the deep level emission in ZnO due to zinc and oxygen vacancy and the energy gap between the interstitials [36,37,38]. Photoluminescence of the as-synthesized ZnO in the blue-green region validate its use as photonic application in blue-green spectral range.

## 4. Conclusions

The present study details the characterization and properties of synthesised ZnO nanoparticles from spent Zn-C battery via thermal nanosizing technique. Synthesized ZnO nanoparticles were spherical in shape, within 50 nm size and confirmed by XRD, XPS, SAED analysis. BET surface area of as-synthesized ZnO nanoparticles were 9.2629 m^2^/g with average BJH pore diameter ~5 nm. UV-Vis spectra showed UV absorbance at around 388 nm wavelength and PL spectra showed visible luminescence. As-synthesized ZnO nanoparticles could be potentially useful for optical applications and will simultaneously provide an economical route to produce ZnO nanoparticles and an effective solution to reduce waste battery in landfills.

## Figures and Tables

**Table 1:** Structural parameters of synthesized ZnO nanoparticles calculated from XRD spectra.

Assigned Miller Indices (hkl)	Calculated d Spacing (nm)	Observed d Spacing (nm)	Particle Size (nm)
100	0.285	0.281	27.93
002	0.262	0.260	28.12
101	0.249	0.248	28.06
110	0.163	0.162	26.43

**Table 2:** XPS results showing the elements with binding energy (BE) and atomic % of the synthesized ZnO nanoparticles.

Name	Start BE	Peak BE	End BE	FWMH eV	Atomic %
O1sA	534.79	530.36	526.69	1.4	26.53
O1sB	534.79	531.56	526.69	1.4	13.63
O1sC	534.79	532.68	526.69	1.39	3.61
C1sA	294.99	284.80	281.39	1.49	6.84
C1sB	294.99	286.47	281.39	1.49	2.12
C1sC	294.99	287.80	281.39	1.49	0.62
C1sD	294.99	289.30	281.39	1.49	1.58
K2p3	294.99	293.32	281.39	1.35	0.31
Ca2p3A	355.09	347.36	343.99	1.94	0.20
Cl2p3A	203.09	198.83	195.79	1.47	5.49
N1sA	404.79	400.90	396.29	1.85	1.79
Na1s	1076.69	1072.26	1068.09	1.74	3.73
Si2p	104.49	101.84	99.09	1.39	3.18
Zn2p3	1026.49	1021.87	1017.49	1.9	29.96

**Table 3:** Surface area analysis of synthesized ZnO nanoparticles.

Name	Results
BET Surface area	9.2629 m^2^/g
Total pore volume of pores at p/p_0_ = 0.95	0.0536 cm^3^/g
Adsorption average pore diameter (4 V/A by BET)	23.17 nm
BJH Adsorption average pore width (4 V/A)	4.93 nm
BJH Desorption average pore width (4 V/A)	4.82 nm

## References

[1] Jayaraman V.K., Álvarez A.M., Amador M.d.l.L.O. (2015). A simple and cost-effective zinc oxide thin film sensor for propane gas detection. Mater. Lett..

[2] İpeksaç T., Kaya F., Kaya C. (2013). Hydrothermal synthesis of zinc oxide (ZnO) nanotubes and its electrophoretic deposition on nickel filter. Mater. Lett..

[3] Khan W., Khan F., Ajmal H.M.S., Huda N.U., Kim J.H., Kim S.-D. (2018). Evolution of structural and optical properties of ZnO nanorods grown on vacuum annealed seed crystallites. Nanomaterials.

[4] Bresser D., Mueller F., Fiedler M., Krueger S., Kloepsch R., Baither D., Winter M., Paillard E., Passerini S. (2013). Transition-metal-doped zinc oxide nanoparticles as a new lithium-ion anode material. Chem. Mater..

[5] Wojnarowicz J., Chudoba T., Gierlotka S., Lojkowski W. (2018). Effect of microwave radiation power on the size of aggregates of ZnO nps prepared using microwave solvothermal synthesis. Nanomaterials.

[6] Giuli G., Trapananti A., Mueller F., Bresser D., d’Acapito F., Passerini S. (2015). Insights into the effect of iron and cobalt doping on the structure of nanosized ZnO. Inorg. Chem..

[7] Jagadish C., Pearton S.J. (2006). Basic Properties and Applications of ZnO, Zinc Oxide Bulk, Thin Films and Nanostructures: Processing, Properties, and Applications.

[8] Bhatte K.D., Sawant D.N., Watile R.A., Bhanage B.M. (2012). A rapid, one step microwave assisted synthesis of nanosize zinc oxide. Mater. Lett..

[9] Khan M.F., Ansari A.H., Hameedullah M., Ahmad E., Husain F.M., Zia Q., Baig U., Zaheer M.R., Alam M.M., Khan A.M. (2016). Sol-gel synthesis of thorn-like ZnO nanoparticles endorsing mechanical stirring effect and their antimicrobial activities: Potential role as nano-antibiotics. Sci. Rep..

[10] Moezzi A., McDonagh A.M., Cortie M.B. (2012). Zinc oxide particles: Synthesis, properties and applications. Chem. Eng. J..

[11] Thirumavalavan M., Huang K.-L., Lee J.-F. (2013). Preparation and morphology studies of nano zinc oxide obtained using native and modified chitosans. Materials.

[12] Zinc Price Surges on Supply Shortage, Renewable Development Keeps Copper on Steady Growth. http://www.Abc.Net.Au/news/2016-08-05/zinc-copper-price-surge/7690040.

[13] Kiddee P., Naidu R., Wong M.H. (2013). Electronic waste management approaches: An overview. Waste Manag..

[14] World Batteries-Demand and Sales Forecasts, Market Share, Market Size, Market Leaders. http://www.Freedoniagroup.Com/world-batteries.html.

[15] (2010). Analysis of Battery Consumption, Recycling and Disposal in Australia. http://www.batteryrecycling.org.au/wp-content/uploads/2011/06/Battery-consumption-recycling-and-disposal-in-Australia_Executive-Summary.pdf.

[16] Ebin B., Petranikova M., Steenari B.-M., Ekberg C. (2016). Production of zinc and manganese oxide particles by pyrolysis of alkaline and Zn–C battery waste. Waste Manag..

[17] Charef S.A., Affoune A., Caballero A., Cruz-Yusta M., Morales J. (2017). Simultaneous recovery of zn and mn from used batteries in acidic and alkaline mediums: A comparative study. Waste Manag..

[18] Deep A., Sharma A.L., Mohanta G.C., Kumar P., Kim K.-H. (2016). A facile chemical route for recovery of high quality zinc oxide nanoparticles from spent alkaline batteries. Waste Manag..

[19] Sobianowska-Turek A., Szczepaniak W., Maciejewski P., Gawlik-Kobylińska M. (2016). Recovery of zinc and manganese, and other metals (Fe, Cu, Ni, Co, Cd, Cr, Na, K) from Zn-MnO_2_ and Zn-C waste batteries: Hydroxyl and carbonate co-precipitation from solution after reducing acidic leaching with use of oxalic acid. J. Power Sources.

[20] Cayumil R., Khanna R., Rajarao R., Mukherjee P.S., Sahajwalla V. (2016). Concentration of precious metals during their recovery from electronic waste. Waste Manag..

[21] Rajagopal R.R., Aravinda L., Rajarao R., Bhat B.R., Sahajwalla V. (2016). Activated carbon derived from non-metallic printed circuit board waste for supercapacitor application. Electrochim. Acta.

[22] Farzana R., Rajarao R., Hassan K., Behera P.R., Sahajwalla V. (2018). Thermal nanosizing: Novel route to synthesize manganese oxide and zinc oxide nanoparticles simultaneously from spent Zn–C battery. J. Clean. Prod..

[23] Rasines I., de Setién J.M. (1980). Thermal analysis of β-Co_2_(OH)_3_Cl and Zn_5_(OH)_5_Cl_2_·H_2_O. Thermochim. Acta.

[24] Mohanan A.A., Parthiban R., Ramakrishnan N. (2015). Alignment nature of ZnO nanowires grown on polished and nanoscale etched lithium niobate surface through self-seeding thermal evaporation method. Materials Res. Bull..

[25] Wang Z.L. (2004). Zinc oxide nanostructures: Growth, properties and applications. J. Phys. Condens. Matter.

[26] Yu D., Trad T., McLeskey J.T., Craciun V., Taylor C.R. (2010). ZnO nanowires synthesized by vapor phase transport deposition on transparent oxide substrates. Nanoscale Res. Lett..

[27] Zhang J., Zhao B., Pan Z., Gu M., Punnoose A. (2015). Synthesis of ZnO nanoparticles with controlled shapes, sizes, aggregations, and surface complex compounds for tuning or switching the photoluminescence. Cryst. Growth Des..

[28] Das J., Pradhan S., Sahu D., Mishra D., Sarangi S., Nayak B., Verma S., Roul B. (2010). Micro-raman and xps studies of pure ZnO ceramics. Phys. B Condens. Matter.

[29] Al-Gaashani R., Radiman S., Daud A.R., Tabet N., Al-Douri Y. (2013). Xps and optical studies of different morphologies of ZnO nanostructures prepared by microwave methods. Ceram. Int..

[30] Herrera-Rivera R., Olvera M., Maldonado A. (2017). Synthesis of ZnO nanopowders by the homogeneous precipitation method: Use of taguchi’s method for analyzing the effect of different variables. J. Nanomater..

[31] Ramimoghadam D., Hussein M.Z.B., Taufiq-Yap Y.H. (2013). Synthesis and characterization of ZnO nanostructures using palm olein as biotemplate. Chem. Cent. J..

[32] Pudukudy M., Hetieqa A., Yaakob Z. (2014). Synthesis, characterization and photocatalytic activity of annealing dependent quasi spherical and capsule like ZnO nanostructures. Appl. Surf. Sci..

[33] Singh R.P.P., Hudiara I., Panday S., Rana S.B. (2016). The effect of Co doping on the structural, optical, and magnetic properties of Fe-doped ZnO nanoparticles. J. Supercond. Novel Magn..

[34] Gogurla N., Sinha A.K., Santra S., Manna S., Ray S.K. (2014). Multifunctional Au-ZnO plasmonic nanostructures for enhanced uv photodetector and room temperature no sensing devices. Sci. Rep..

[35] Talam S., Karumuri S.R., Gunnam N. (2012). Synthesis, characterization, and spectroscopic properties of ZnO nanoparticles. ISRN Nanotechnol..

[36] Djurišić A.B., Leung Y.H. (2006). Optical properties of ZnO nanostructures. Small.

[37] Feng Y., Zhou Y., Liu Y., Zhang G., Zhang X. (2006). Photoluminescence spectra of nano-structured ZnO thin films. J. Lumin..

[38] Nagaraju G., Nagabhushana H., Suresh D., Anupama C., Raghu G., Sharma S. (2017). Vitis labruska skin extract assisted green synthesis of ZnO super structures for multifunctional applications. Ceram. Int..

